# Anhedonia to music and mu-opioids: Evidence from the administration of naltrexone

**DOI:** 10.1038/srep41952

**Published:** 2017-02-08

**Authors:** Adiel Mallik, Mona Lisa Chanda, Daniel J. Levitin

**Affiliations:** 1 Integrated Program in Neuroscience, McGill University, Canada; 2 Department of Psychology, McGill University, Canada; 3 School of Computer Science and School of Music, McGill University, Canada.

## Abstract

Music’s universality and its ability to deeply affect emotions suggest an evolutionary origin. Previous investigators have found that naltrexone (NTX), a μ-opioid antagonist, may induce reversible anhedonia, attenuating both positive and negative emotions. The neurochemical basis of musical experience is not well-understood, and the NTX-induced anhedonia hypothesis has not been tested with music. Accordingly, we administered NTX or placebo on two different days in a double-blind crossover study, and assessed participants’ responses to music using both psychophysiological (objective) and behavioral (subjective) measures. We found that both positive and negative emotions were attenuated. We conclude that endogenous opioids are critical to experiencing both positive and negative emotions in music, and that music uses the same reward pathways as food, drug and sexual pleasure. Our findings add to the growing body of evidence for the evolutionary biological substrates of music.

Music is a human universal — no known culture now or any time in the past has lacked music^1^,^2^, and its emotional significance is well known. Mothers in every culture sing to their infants, making music one of the newborn’s first experiences^3^. It is present during a wide variety of human activities — birthdays, religious ceremonies, political gatherings, sporting events, parties and romantic encounters. Although the neural underpinnings of music cognition have been widely studied in the last fifteen years, relatively little is known about the neurochemical processes underlying musical pleasure^4^.

Preliminary studies have shown that music listening and performing modulate levels of serotonin^5^, epinepherine, dopamine, oxytocin, and prolactin^4^. Music can reliably induce feelings of pleasure^6^, and indeed, people consistently rank music as among the top ten things in their lives that bring pleasure, above money, food and art^7^.

Reward is experienced in two phases^8^,^9^, with distinct neurochemical correlates: an appetitive/anticipatory phase, driven by the mesotelencephalic dopaminergic pathway, and a consummatory phase, driven by both dopamine and μ-opioid receptor activation^9^,^10^,^11^,^12^. Anticipatory and consummatory pleasure — wanting and liking, respectively — depend on different sites within the nucleus accumbens (NAcc) Anticipatory pleasure is linked to a widely distributed network throughout the NAcc, whereas consummatory pleasure is linked to the rostro-dorsal quarter of the medial accumbens shell^13^. Pleasurable music has been shown in several studies to activate the NAcc^6^,^14^,^15^.

The opioid and dopaminergic systems are anatomically linked and previous studies have shown that blocking the opioid system can reduce dopaminergic activity^16^,^17^,^18^,^19^,^20^,^21^,^22^. That is, if one pharmaceutically blocks the opioid-mediated consummatory reward circuits, dopamine-mediated anticipatory reward circuits are likely to be affected simultaneously.

Naltrexone (NTX) and the chemically similar naloxone are broad-spectrum antagonists that block μ-opioid, kappa and (to a lesser extent, 10:1) delta opioid receptors. They have been shown to reduce reward after physical activity^23^,^24^, reduce food pleasantness and subjective appetite during eating^25^,^26^ and modulate pain and mood^27^. For the past few decades, they have been used to treat alcohol and opioid dependence^28^, and to reduce consumption of pleasurable foods in rats^29^,^30^ and humans^31^,^32^. An intriguing finding is that NTX administration is associated with a generalized reduction in hedonics — participants report feeling reduced highs and lows, joy and sadness^33^,^34^,^35^. This informed the present study, in which we sought to investigate musical anhedonia — to explore whether musical emotion is governed by the same hedonic/reward system as other experiences, such as food, drugs, and sex.

Previous investigators thought that the action of naloxone and NTX was specific to food and drink reward^25^,^26^,^32^, and did not extend to sex^36^,^37^,^38^ or music^39^. To date, only one study has directly explored the relation between opioids and music. A small group of participants received placebo or naloxone in a between-subjects design and experimenters gauged their musical pleasure through self-report of musical “chills”^40^. Three of the ten participants reported reduced musical pleasure in the naloxone condition, although data on the remaining seven participants were not reported. No objective measures were collected.

We administered NTX to a group of naïve participants in a double-blind placebo controlled experiment, and assessed musical reward using a combination of objective and subjective measures. Further, we controlled for musical experience, state and trait anxiety, arousal, and the familiarity of the music. We instructed participants to choose their own favorite piece of music, and we also presented experimentally verified “neutral” music (neither happy nor sad) as a control for emotional valence and intensity.

Our principal hypothesis was that NTX would produce anhedonia, reducing both positive and negative reactions to music. A subsidiary hypothesis was that these effects would be greater for self-selected pleasurable music than for experimenter-selected neutral music, because the former stimulus has more emotion to attenuate. The protocol received approval from Health Canada and the McGill University Institutional Review Board for Medicine, and was conducted in accordance with the ethical principles stated in the Declaration of Helsinki^41^.

## Results

Participants were asked to bring to the laboratory two music recordings that reliably produced intense feelings of pleasure for them, including but not limited to the sensation of chills. Self-selection has been found to be a reliable means of eliciting an emotional response to music in participants, while providing experimental control for their emotional reactions — the acoustic stimulus may differ across participants, but its emotion-stimulating properties do not vary as much as they might if the experimenter imposed a single musical selection on all participants^42^,^43^,^44^. We placed no restrictions on stylistic or structural aspects of the music, given that these factors have not significantly contributed to the pleasurable effects of music in previous studies^45^. Two pieces of music, previously experimentally verified as emotionally neutral, were used for the neutral music condition^46^. Participants listened to all music on a computer with headphones at a volume they adjusted for their comfort, typically 60–70 dB(A).

We collected continuous measures of both objective and subjective pleasure. The objective measures were obtained using the Biopac MP35 system (Biopac Systems, Inc., Goleta, CA) to obtain electromyograms of the zygomatic (smile) and corrugator (frown) facial muscles. For the subjective measure, participants controlled a MIDI slider in real-time on a 100-point scale (these were subsequently scaled to [0,10]; see ref. 47 for additional details of methods). To account for any generalized behavioral and physiological effects of the NTX we administered the state trait anxiety inventory (STAI), profile of mood states (POMS), zygomatic (EMG ZYG) and corrugator (EMG COR) electromyograms, blood volume pulse amplitude (BVPA), and respiratory rate (RR) pre- and post-administration of drug or placebo, and found no significant differences (Figure S4).

**Figure 1:**
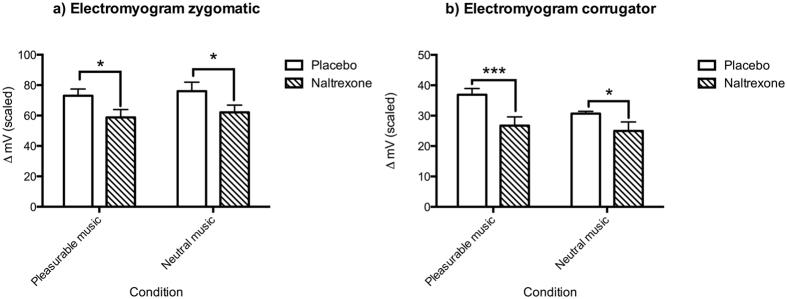
Opioid blockade (Naltrexone condition, NTX) caused a decrease in (**a**) zygomatic and (**b**) corrugator muscle activity (*denotes *p* < 0.05, ***denotes *p* < 0.001). Error bars indicate standard error of mean. Precise *p* values given in text.

Our first hypothesis was that the opioid blockade would reduce both positive and negative valence emotional reactions, as indicated by reductions in the zygomatic and corrugator muscle responses, respectively. This was confirmed (Fig. 1) by Fisher’s Randomization Resampling Test (FRT, one-tailed)^48^, showing significant reductions in muscle activity in the drug condition. We report the p value of the FRT followed by the effect size (as measured by Cohen’s *d*, the normalized difference between two means). For the EMG ZYG, comparing placebo to NTX, *p* = 0.02 (d = 0.77) for pleasurable music, *p* = 0.03 (d = 0.67) for neutral music; for the EMG COR, *p* = 0.001 (d = 1.05) for pleasurable music, *p* = 0.04 (d = 0.69) for neutral music.

We obtained the following means (±standard error of the mean in scaled units). For the EMG ZYG (Fig. 1a), pleasurable music placebo = 73.07 (4.38); pleasurable music NTX = 58.76 (5.48); neutral music placebo = 58.76 (5.17); neutral music NTX = 76.03 (5.96). For the EMG COR (Fig. 1b), pleasurable music placebo = 36.88 (2.07); pleasurable music NTX = 26.74 (2.86), neutral music placebo = 30.69 (0.73); neutral music NTX = 24.98 (2.95).

**Figure 2:**
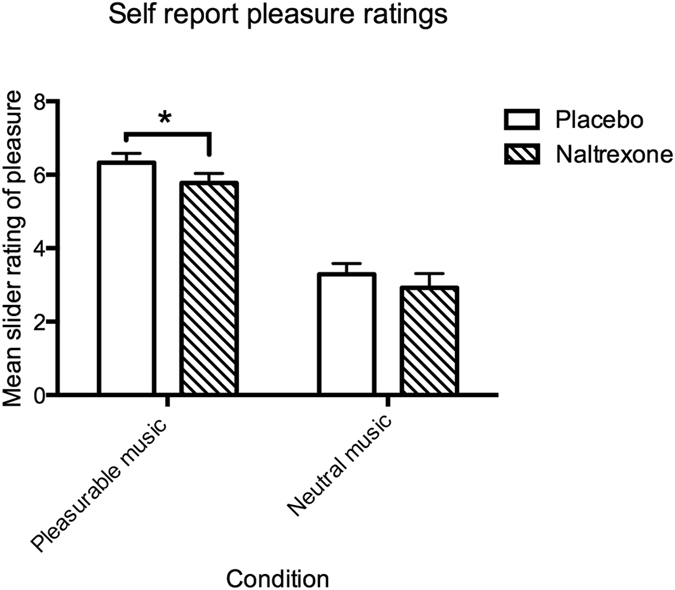
Effects of opioid blockade (Naltrexone condition, NTX). NTX caused a decrease in self-report measures of pleasure for pleasurable music (*p* < 0.05) but not neutral music, (*p* = 0.12). Error bars indicate standard error of mean.

Our second hypothesis was that the opioid blockade would cause subjective measures of pleasure/reward to decrease, relative to the placebo condition, in parallel to the physiological EMG results. This would provide additional evidence that endogenous opioids moderate musical reward, as they do for food, drug and sex pleasure. This was confirmed by t-test on the real-time slider ratings for the pleasurable music condition (Fig. 2). Comparing placebo to NTX, t = 1.72, *p* < 0.05 (d = 0.50), and by FRT, *p* = 0.000. As one might expect, because neutral music is already attenuated, the comparison was not significant, t = 1.22, *p* = 0.12 (d = 0.13) for neutral music.

We obtained the following means (±standard error of the mean in scaled units) for the slider ratings (Fig. 2). Pleasurable music placebo = 6.33 (0.25); pleasurable music NTX = 5.78 (0.26); neutral music placebo = 3.29(0.29); neutral music NTX = 2.92 (0.38).

A subsidiary hypothesis was that the effects of NTX would be larger on the participants’ self-chosen, pleasurable music than on neutral music, because the pleasurable music should have more emotion to attenuate. Indeed the effects sizes above confirmed this to be true for both the EMG ZYG and EMG COR and for the real-time slider ratings.

As an exploratory measure, we collected additional data from the BioPac on four dependent variables: blood volume pulse amplitude (BVPA), respiratory rate (RR), heart rate (HR) and galvanic skin response (GSR). The GSR data were noisy due to motion artifacts and so we discarded them. We compared area under the curve for the duration of the experiment, as well as mean and median values, using FRT and found no statistical significance for the comparison of NTX versus placebo for the remaining three dependent variables. However, one can consider the five variates we collected with the Biopac as separate experiments: if the placebo and NTX conditions truly didn’t differ, that is, if they are just arbitrary labels attached to columns of data, we would expect a roughly even distribution of the direction effects. To the contrary, we found that all five dependent variables showed effects in the hypothesized direction, and by the sign test, this yields p = 0.03. This provides additional evidence for the NTX-induced anhedonia hypothesis.

## Discussion

Our primary hypothesis was based on the previous literature which illustrated the role of endogenous opioids in mediating food, drug, and sex pleasure^25^,^26^,^35^,^37^,^49^,^50^; we predicted that blocking μ-opioid receptors with NTX would cause decreased physiological reactions to music for both positive and negative emotions. This was confirmed for both pleasurable and neutral music, compared to placebo, through EMG recordings of two different facial muscles (Fig. 1), supporting the NTX-induced anhedonia hypothesis.

Our secondary hypothesis was that these findings would be replicated with subjective, self-report measures of ongoing, real-time musical pleasure. This was confirmed for pleasurable music, but not neutral music. One might expect this, given that the neutral music is already attenuated in the two dimensions of valence and intensity. Here we face an inconsistency between the physiological and subjective measures. One possibility is that the physiological measures for neutral music have lower variability than the subjective measures (lower variability increases the likelihood of detecting statistical differences that exist). We confirmed this by computing coefficients of variation (CV), which were lower for the EMG measures than for the slider measure. For placebo, slider *CV* = 0.36, EMG ZYG *CV* = 0.34 and EMG COR *CV* = 0.09. For NTX, slider *CV* = 0.57, EMG ZYG *CV* = 0.30, EMG COR *CV* = 0.46.

Our subsidiary hypothesis was that the effects of the μ-opioid blockade would be greater for pleasurable music (because of its higher levels of emotional intensity and valence) than neutral music. This was confirmed for all three of our dependent measures. In addition, we collected supplementary psychobiological measures, BVPA, RR, and HR. Although none of them showed a main effect themselves, we adopted a meta-analytic approach and found that all three showed an effect in the hypothesized direction (p = 0.13) and, when taken together with the EMG measures, all five showed an effect in the hypothesized direction (p = 0.03).

Here, we’ve provided evidence for the NTX-induced anhedonia hypothesis, and thus evidence that musical pleasure is mediated by the brain’s endogenous opioid system. The fact that music listening triggers a well-defined neurochemical response suggests an evolutionary origin for music, although one must be cautious and not over-interpret these results; it is also possible that music has developed to exploit an already existing reward system that evolved for other purposes, such as recognizing and responding appropriately to various human and animal vocalizations.

The current experimental finding of reduced response from both the positive and negative valence EMGs (ZYG and COR respectively) reinforces the notion that music is complex and rarely conveys a single emotional valence. Listeners more often report finding music to be bittersweet than purely happy or purely sad^51^,^52^, and many listeners report that even sad music brings them pleasure^53^. This may appear to contradict some of the literature with regard to opioids, pain relief and induced sadness. Prior work showed that pain and induced sadness reduce *μ*-opioid transmission as reflected by an increase in *μ*-opioid receptor availability *in vivo* in an induced sadness condition (participants recalled a tragic autobiographical event) compared to a neutral mood condition^54^,^55^. However, sadness evoked by music is different from sadness induced by pain or by recalling a tragic event. According to Levinson^56^, some of the qualitative rewards of sad music include savoring the feeling, understanding the feeling, reassuring oneself of the ability to feel intense emotions and sharing the sadness with others (in many cases the composer). These rewards are not directed to any real-life circumstances and thus tend not to evoke the negative aspects of sadness (i.e. grieving over loss of a loved one), which in turn may not elicit the neurochemical response of sadness and depression caused by tragic events. To a certain extent this was verified by Panksepp^57^ who showed that sad music evokes more intense physiologically pleasurable responses (“chills”) than happy music.

Our administration of 50 mg NTX is known to block up to 80% of the μ-opioid and some δ-opioid receptors^58^; this underlies the observed decrease in pleasure relative to the placebo condition. An additional finding of interest in the EMG COR is found when neutral music condition is subtracted from that of pleasurable music condition for both placebo and NTX groups. We see a significant decrease in the difference between pleasurable and neutral music in the EMG COR (Figure S2) for the NTX group relative to the placebo group. This smaller difference further shows that NTX decreases the range of emotional response to music stimuli and adds to the weight of evidence that opioids don’t merely sub-serve pleasure and the positive valence emotions, but are implicated in any emotional changes, positive or negative^35^,^37^,^50^.

One limitation in this study is the inability to produce a double-dissociation between anticipatory (dopamine-mediated) and consummatory (opioid-mediated) pleasure, primarily because no selective dopamine antagonist exists that is safe for human administration. A second limitation is intrinsic. Music unfolds over time, and creates ongoing expectations. Although we selectively blocked the consummatory pleasure system, the pleasure phases play out differently in music than in, say, drugs, sex, and food, when the fulfillment of expectations is more well-defined in time. Anticipatory pleasures may exist during the consumption phase of music listening as new musical structures are introduced that build up ongoing expectation and release patterns. In fact, in a prior study, we demonstrated that musical anticipation is intimately bound with fulfillment of pleasure by showing that brain activity in the default mode network (as indexed by the BOLD response in fMRI) reaches a *maximum* during periods of silence, when no music is playing at section transitions in symphonic works^59^.

Further supporting the blurred distinction between anticipation and consumption reward in music, a previous study^15^ using positron emission tomography (PET) with the selective dopamine radiotracer [^11^C] raclopride found dopamine release during both anticipatory and consummatory phases of music listening, but in different neural networks. Dopamine may therefore mediate pleasure in both cases, and opioids could be acting in a more global fashion, as evidenced by the attenuation of both sadness and happiness that we found. Supporting this notion is that the μ-opioid and dopaminergic systems are linked: blocking the μ-opioid receptors in rats in the ventral tegmental area decreases dopamine levels as these μ-opioid receptors provide stimulation to dopaminergic neurons which project to the nucleus accumbens^16^,^17^,^18^,^19^,^20^,^22^.

Another limitation is the generalizability of our study. As is typically done in behavioral research, we selected participants from a university community and regardless of the statistical tests used, one should not assume that such a restricted population is representative of the general population.

We have shown here that the opioid system is responsible for mediating the elicitations of both positive (pleasurable) and negative (sadness) emotional responses to music. It remains to be seen exactly how the opioid system interacts with the dopaminergic system in emotional responses to music. Future studies using music stimuli in which anticipatory expectations are violated or computer generated music in which only the anticipatory component is included could be used to more clearly differentiate the anticipatory phase from the consumption phase in music^60^,^61^. In addition, future PET studies using both dopamine and opioid radiotracers can further shed light on how these two systems interact.

## Materials and Methods

### Participants

Participants were recruited from advertisements placed around McGill University and on the web. Twenty-one healthy adult participants met our screening criteria (detailed in the Supplementary Material) and were included in the study. Three male and one female participant experienced adverse reactions to the naltrexone (NTX) and withdrew from the study, yielding seventeen participants (7 male, 10 female). Equipment errors occurred with two participants, reducing the dataset to fifteen participants (6 male, 9 female). Participants were paid $100 for their participation, plus up to $40 for transportation and/or parking. The experiment took approximately 90 minutes on each of two separate days one week apart (to provide a wash-out period for the drug).

## Design

This was a double-blind, placebo-controlled, randomized cross-over study in order to test the effects of the following:

1. *Drug Condition*: (a) NTX or (b) placebo.
2. *Stimuli*: (a) 2 pieces of self-selected pleasurable music and (b) 2 pieces of experimenter-selected neutral music.

Dependent measures collected continuously during stimulus presentation were:

1. Real-time recordings of subjective pleasure states (scale: 0–100) using a MIDI slider interfaced with a computer^58^.
2. Electromyograms of the zygomatic (EMG ZYG) and
3. Corrugator (EMG COR) facial muscles.

These were compared to baseline measurements taken upon arrival at the lab and again one hour following drug admistration. Mean, median and area under the curve statistics for all aforementioned dependent measures were analyzed. These measures were averaged across the duration of each stimulus (song) regardless of stimulus (song) duration.

## Procedure

On arrival at the laboratory, and after obtaining informed consent, participants confirmed they met the pre-conditions of the study (e.g. no alcohol within 24 hours, no drug use within 10 days; not pregnant). We verified this with urine tests in the laboratory (additional details in the Supplementary Materials). We outfitted participants with Biopac sensors (Biopac Systems, Goleta, CA) and instructed them in the use of the real-time slider scale, and obtained a five-minute baseline (pre-drug) of physiological measures. We then administered drug or placebo in a double-blind counterbalanced fashion, according to a pre-arranged order from a random number generator. During the one hour between drug administration and peak bioavailability, participants filled out questionnaires for an unrelated study and filled any remaining time by reading materials they brought with them (mostly homework). This preparation period allowed us to keep participants isolated from any music for one hour prior to testing.

One hour post-drug or placebo administration, participants were seated at the computer, and we obtained a five-minute baseline (post-drug) of physiological measures. Participants then listened to the two pieces of self-selected, self-identified pleasurable music they had provided, and two pieces of experimentally neutral music, while making real-time slider ratings of their ongoing pleasure. They listened to music over studio-quality headphones (Sennheiser HD-600, Wedemark, Germany) as digitized full bandwidth (.aiff, 44.1 Khz, 16-bit) files played back through iTunes on a computer (Apple MacIntosh G5, Cupertino, CA) at a volume they adjusted for their comfort, typically 62–72 dB(A).

Following the conclusion of the experiment, participants completed a post-experiment survey. Participants returned to the lab one week later for the complementary drug/placebo condition following identical procedures.

## Pharmacological agent

We administered naltrexone hydrochloride (Revia®, Bristol-Myers Squibb) or placebo in a double-blind, counterbalanced fashion (i.e. NTX on Day 1, placebo on day 2, or the reverse order). Day 1 and Day 2 were spaced 7 days apart to allow a drug wash-out period between tests^62^. To ensure double-blind conditions, a compounding pharmacy (Paylan Pharmacy, Montreal, QC) prepared the naltrexone hydrochloride in an opaque blue, unmarked capsule with cellulose filler, and used identical opaque blue, unmarked capsules with identical cellulose filler as controls. An independent set of judges was unable to tell the capsules apart by inspection. We administered 50 mg NTX, based on previous findings that this dose effectively blocked the pleasantness of highly palatable foods (e.g. sweetened or fatty foods) without affecting taste perception^25^,^26^. This dosage is known to block up to 80% of μ-opioid and some Δ-opioid receptors^58^ and is the lowest effective dose found in the literature. Higher doses may have yielded greater effects sizes, but we sought to obtain effects at the lowest possible dose to minimize the chance of harm to our participants. Testing occurred 1-hour post drug administration, when peak plasma concentrations and maximum blockade of central μ-opioid receptors are attained^25^,^26^,^63^. Clinical studies have found NTX to be generally well tolerated in healthy individuals^63^.

### Musical stimuli

Participants were asked to bring to the laboratory two music recordings that reliably produced intense feelings of pleasure for them, including chills. Self-selection has been previously found to be a reliable means of eliciting an emotional response to music in participants^43^,^44^. There were no restrictions placed on stylistic or structural aspects of the music, given that these factors have not significantly contributed to the pleasurable effects of music in previous studies^45^. Two pieces of music that were previously experimentally verified as neutral were used for the neutral music condition^46^. Participants listened to all music on a computer (PowerMac G5, Apple Computer) with headphones (Sennheiser HD600, Wedemark, Germany) at a volume they adjusted for their comfort, typically 60–70 dB(A).

### Physiological measurements

We collected physiological baseline data from participants over a 5-minute silent relaxation period at two different time-points: immediately before drug/placebo administration (25 minute after arrival in the laboratory) and 1-hour post-drug/placebo (immediately before the stimuli are presented). We collected experimental physiological data from participants during presentation of the musical stimuli, using the Biopac MP35 system, and following the procedures of^64^. BSL Pro 3.7.3 software (Biopac Systems, Inc., Goleta, CA) was used to collect the Biopac data. A photoplethysmyograph sensor was attached on the middle finger for recording (BVPA), bipolar miniature (Ag/AgCl) skin electrodes were placed on the corrugator supercilii and zygomaticus major muscles regions in the left side of the participants face as mentioned previously^65^. A respiration sensor was placed around the diaphragm to record the respiratory rate of the participant.

### Data analysis

#### Behavioral self-report measures

According to the D’Agostino and Pearson omnibus normality test, our data were not normally distributed. Thus, data were analyzed with Fisher’s Randomization Resampling Test (FRT, 2000 iterations^48^), also known as a permutation test or Monte Carlo method, between the NTX and placebo groups in each stimulus condition (pleasure music and neutral music). FRT is a good way to control the type I error rate for multiple comparisons^66^, it is non-parametric, and so makes no assumptions about the underlying distribution of the data that are common in other inferential statistical tests^48^,^66^,^67^,^68^. We performed one-tailed tests based on our a priori assumption that ratings for the NTX condition would be attenuated relative to those for the placebo condition. We also conducted one-tailed Fisher randomization tests (2000 iterations) between the pleasurable music and neutral music conditions in both the placebo and NTX group as we hypothesized that the pleasurable music condition will have a higher pleasurable rating and physiological response compared to the neutral music condition.

### Physiological dependent measures

We obtained two baselines for EMG ZYG, EMG COR, BVPA and RR: prior to drug/placebo administration, and one-hour post drug/placebo administration. We compared the differences to assess whether NTX had any impact on these physiological measures (Figure S4). The data failed the D’Agostino and Pearson omnibus normality test and we thus conducted FRTs. We found that the NTX did significantly impact these measures (Figure S4), although we note trends for BVPA and EMG ZYG – for BVPA, NTX tended toward reduced response, whereas for EMG ZYG NTX tended toward increased response. To assess the main effects of EMG ZYG and EMG COR, given no differences between the baseline 2 and baseline 1 values, we subtracted baseline 2 measures from the experimental measurements taken during stimulus presentation on a subject-by-subject basis. Given our prior hypothesis of the NTX group having a lower pleasurable rating and physiological response, we then conducted one-tailed Fisher randomization tests (2000 iterations) between the NTX and placebo groups in each condition (pleasurable music, and neutral music). We also conducted one-tailed Fisher randomization tests (2000 iterations) between pleasurable music and neutral music conditions in the placebo and NTX group because we hypothesized that the pleasurable music condition will have a higher pleasurable rating and physiological response compared with the neutral music condition.

## Additional Information

**How to cite this article:** Mallik, A. *et al*. Anhedonia to music and mu-opioids: Evidence from the administration of naltrexone. *Sci. Rep.* **7**, 41952; doi: 10.1038/srep41952 (2017).

**Publisher's note:** Springer Nature remains neutral with regard to jurisdictional claims in published maps and institutional affiliations.

## Supplementary Material

Supplementary Information
