# Supplementary material for: Anhedonia to music and mu-opioids: Evidence from the administration of naltrexone
Source: Sci Rep. 2017 Feb 8;7:41952. doi: 10.1038/srep41952 (PMC5296903; doi:10.1038/srep41952)
Supplement: Supplementary Information [file srep41952-s1.pdf]

Supplementary materials to Anhedonia to music and mu-opioids: evidence from the administration of naltrexone

Adiel Mallik[1], Mona Lisa Chanda[2], and Daniel J. Levitin[1,2,3]

Participant selection and screening.

Applicants were screened for normal hearing and health status. Exclusionary criteria were an allergy to naltrexone, kidney or liver injury or disorder, bipolar, panic or psychotic disorders, epilepsy, smoking more than 15 cigarettes per day, pregnancy, substance abuse, use of opioid analgesics, cocaine, recreational drugs (e.g. marijuana, LSD, ecstasy, etc.) and prescription medication (except oral contraceptives) within the past 10 days, use of over-the-counter drugs (e.g. analgesics, anti-inflammatories, sleeping aids, etc.) or alcohol within the past 24 hours. Participants were also excluded if they reported they were currently in pain (e.g. headache), or if they had used anti-diarrheal medications in the seven days prior to the study (these are known to interact with naltrexone, e.g. Immodium, Kaopectate, Pepto-Bismol). Because naltrexone is metabolized by the liver and kidneys [1-3], participants were required to provide medical records reporting normal kidney and liver function results within the past year. We administered blood tests through an independent laboratory (Medisys, Montreal, QC) for kidney and liver function for potential participants if they had not already done so in the past year and wanted to be included; we compensated them $15 for this. Individuals with evidence of abnormal kidney or liver function were excluded from the study.

All participants who passed initial kidney and liver screening were scheduled for participation in the experiment, and completed further screening on the two days of testing for drug use and pregnancy. Screening for drugs of abuse was conducted with a DrugCheck® Urine Drug Test - 5 panel (DTK, Barrie, ON). The test detects cocaine, opiates, δ^9-tetrahydrocannabinol, amphetamine and methamphetamine. Urine pregnancy screening was conducted with BFP hCG test strips (Fairhaven Health, Bellingham, WA, USA) *in vitro* test. There were no positive results on these tests and thus no participants were excluded at this stage.

Sample size and justification.

Based on a power analysis with a specified effect size Cohen's $f = 0.9$, $\alpha = 0.05$ and power $(1-\beta) = 0.70$, we required a sample size of $n = 15$ participants. Two participants withdrew due to naltrexone side effects and were replaced, yielding the desired total of 15 participants. Equipment failures occurred with two participants during the physiological measures, reducing the physiological dataset to fifteen participants.

Data Analysis

We had two cases of missing data due to equipment malfunction, and as such, these values were treated as missing completely at random (MCAR) data, and we used mean imputation to replace them [4].

Choice of naltrexone over naloxone.

We chose to administer naltrexone rather than naloxone for the following reasons: naltrexone can be given orally, in pill form, whereas naloxone must be

administered intravenously. The half-life of naltrexone is 4 hours and of naloxone is 60 minutes (which may not have been long enough for us to properly conduct the study, which was estimated to take 1.5 – 2 hours). The two substances are chemically similar, both are μ-opioid antagonists, and are used in similar studies in the research literature (Figure S1).

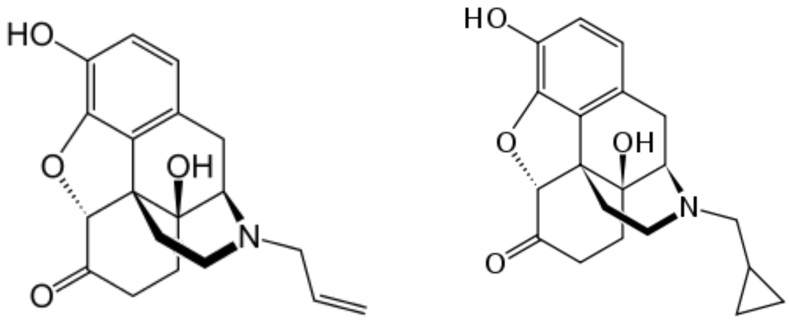

Figure S1.

(a) Naloxone molecule (b) Naltrexone molecule

Supplementary results

Physiological measures: *pleasure and neutral music difference of electromyograms of corrugator muscles*

The naltrexone group revealed a significantly smaller difference between pleasurable music and neutral music in area under the curve electromyogram corrugator measurements by FRT (Figure S2). For the zygomatic electromyogram there were no significant differences between pleasurable and neutral music for either the placebo (p = 0.23) or naltrexone conditions (p = 0.29).

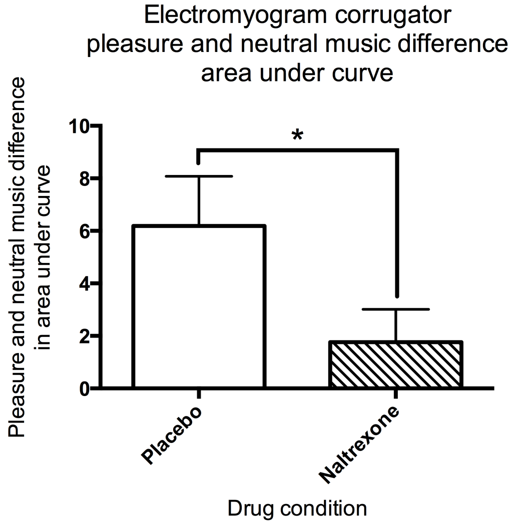

Figure S2. Opioid blockade (Naltrexone condition, NTX) caused a decrease in the difference between pleasurable and neutral music in the corrugator electromyogram ($p < 0.05$). Exact one tailed p value comparing placebo and naltrexone is: $p = 0.015$. Mean ± standard error of mean (SEM) in scaled units were placebo (6.19 ± 1.90), naltrexone (1.76 ± 1.25).

Behavioral measures: *changes in state trait anxiety inventory (STAI) and profile of mood states (POMS) after placebo/naltrexone treatment*

The opioid blockade had no statistically significant impact on anxiety or mood according to our administration of the STAI and POMS questionnaires 1 hour after placebo/drug treatment, which suggests that the naltrexone did not significantly affect the baseline mood of the participant.

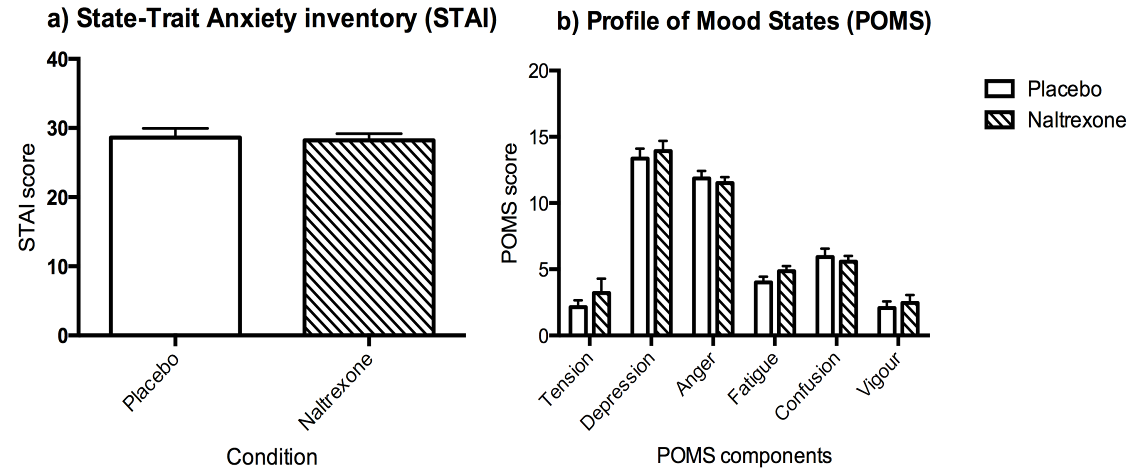

Figure S3. Opioid blockade showed no significant changes in a) State-Trait Anxiety Inventory (STAI) or in any of the b) Profile of Mood States (POMS) components. Error bars indicate standard error of mean. Exact one tailed p values comparing placebo and naltrexone are: a) STAI score: p = 0.39, b) Tension: p = 0.13, depression: p = 0.25, anger: p = 0.30, fatigue: p = 0.08, confusion: p = 0.23 and vigor: p = 0.31. Mean ± standard error of mean (SEM) are a) placebo: 28.62 ± 1.35, naltrexone: 28.23 ± 0.96. b) tension placebo: 2.14 ± 0.52, tension naltrexone: 3.21 ± 1.08, depression placebo: 13.36 ± 0.75, depression naltrexone: 13.93 ± 0.76, anger placebo: 11.86 ± 0.57 , anger naltrexone: 11.50 ± 0.45, fatigue placebo: 4.0 ± 0.44, fatigue naltrexone: 4.86 ± 0.39 , confusion placebo: 5.94 ± 0.62, confusion naltrexone: 5.57 ± 0.44, vigour placebo: 2.08 ± 0.50, vigour naltrexone: 2.46 ± 0.60.

Physiological measures: *changes in differences between post-placebo/drug and pre-placebo/drug for BVPA, RR, EMG ZYG and EMG COR.*

The pre-placebo/drug baseline was subtracted from the post-placebo/drug baseline to see if the naltrexone had a significant impact on physiological measures (BVPA, RR, EMG ZYG and EMG COR) after administration compared to the placebo. The placebo pre/post baseline difference was not found to be significantly different

that the naltrexone pre/post baseline difference for all physiological measures (BVPA, RR, EMG ZYG, EMG COR) (Figure S4) indicating that the naltrexone did not have a statistically significant impact on baseline physiological measures (BVPA, RR, EMG ZYG, EMG COR).

Post-placebo/drug minus pre-placebo/drug physiological baselines

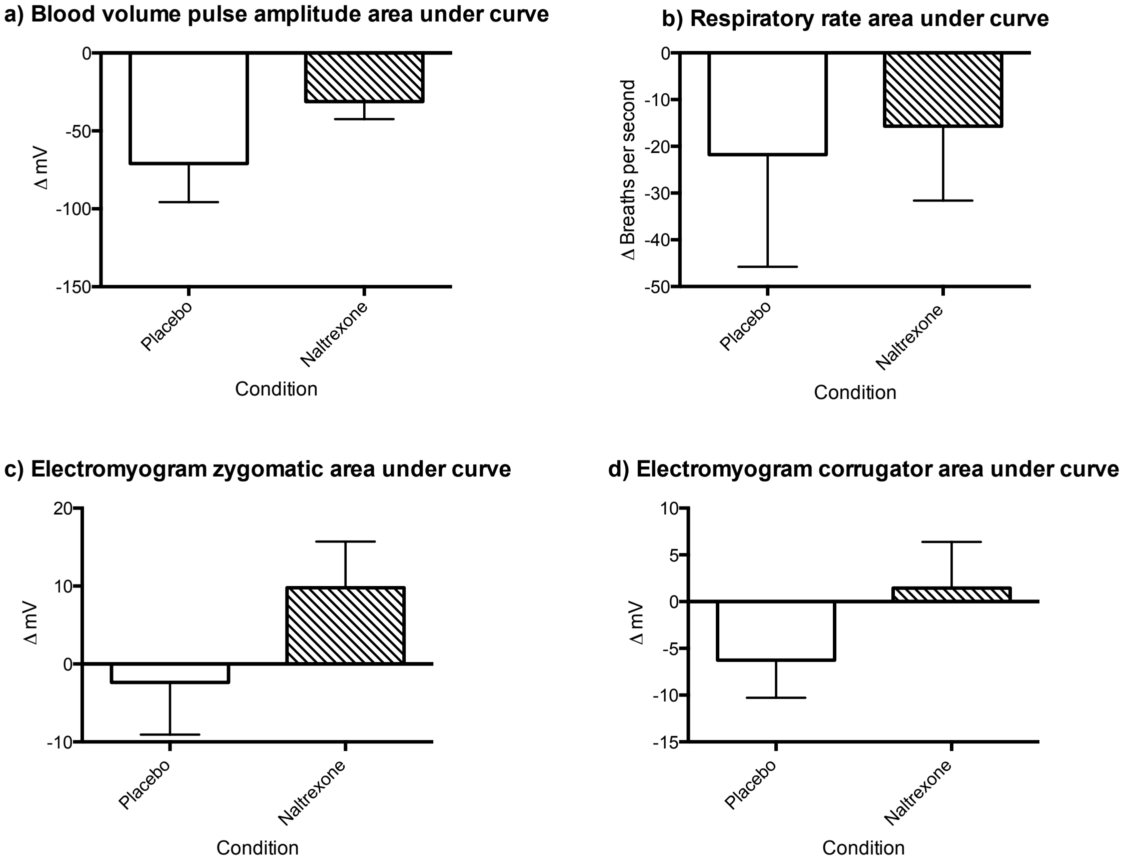

Figure S4. Opioid blockade showed no significant changes in differences between pre and post placebo/drug baselines in a) BVPA, b) RR, c) EMG ZYG, d) EMG COR. Error bars indicate standard error of mean. Exact one tailed p values comparing placebo and naltrexone are: a) BVPA p = 0.10, b) RR p = 0.42, c) EMG ZYG p = 0.07, d) EMG COR p = 0.15. Mean ± standard error of mean (SEM) are: a) BVPA placebo: -70.98 ± 24.76, BVPA naltrexone: -31.15 ± 11.35, b) RR placebo: -21.76 ± 24.02, RR naltrexone: -

15.67 ± 15.91, c) EMG ZYG placebo: -2.35 ± 6.72, EMG ZYG naltrexone: 9.78 ± 5.94, d) EMG COR placebo: -6.26 ± 4.01, EMG COR naltrexone: 1.46 ± 4.94.

Supplementary Materials References

1 Crabtree, B. Review of naltrexone, a long-acting opiate antagonist. *Clinical Pharmacy* **3**, 273-280 (1984).

2 Verebey, K., Volavka, J., Mulé, S. & Resnick, R. Naltrexone: disposition, metabolism, and effects after acute and chronic dosing. *Clinical Pharmacology and Therapeutics* **20**, 315-328 (1976).

3 Wall, M., Brine, D. & Perez-Reyes, M. The metabolism of naltrexone in man. *NIDA Research Monograph* **28**, 105-131 (1980).

4 Ali, A. M. G., Dawson, S. J., Blows, F. M., Provenzano, E., Ellis, I. O., Baglietto, L., ... & Pharoah, P. D. Comparison of methods for handling missing data on immunohistochemical markers in survival analysis of breast cancer. *British Journal of Cancer*, **104**, 693-699 (2011).
